# Genetic analysis by targeted next-generation sequencing and novel variation identification of maple syrup urine disease in Chinese Han population

**DOI:** 10.1038/s41598-021-98357-2

**Published:** 2021-09-23

**Authors:** Xiaohua Fang, Xiaofan Zhu, Yin Feng, Ying Bai, Xuechao Zhao, Ning Liu, Xiangdong Kong

**Affiliations:** Obstetrics and Gynecology Department, Genetics and Prenatal Diagnosis Center, The First Affiliated Hospital of Zhengzhou University, Zhengzhou, 450052 People’s Republic of China

**Keywords:** Biological techniques, Biotechnology, Computational biology and bioinformatics, Genetics, Molecular biology, Structural biology, Diseases, Medical research, Molecular medicine

## Abstract

Maple syrup urine disease (MSUD) is a rare autosomal recessive disorder that affects the degradation of branched chain amino acids (BCAAs). Only a few cases of MSUD have been documented in Mainland China. In this report, 8 patients (4 females and 4 males) with MSUD from 8 unrelated Chinese Han families were diagnosed at the age of 6 days to 4 months. All the coding regions and exon/intron boundaries of *BCKDHA*, *BCDKHB*, *DBT* and *DLD* genes were analyzed by targeted NGS in the 8 MSUD pedigrees. Targeted NGS revealed 2 pedigrees with MSUD Ia, 5 pedigrees with Ib, 1 pedigree with MSUD II. Totally, 13 variants were detected, including 2 variants (p.Ala216Val and p.Gly281Arg) in *BCKDHA* gene, 10 variants (p.Gly95Ala, p.Ser171Pro, p.Phe175Leu, p.Arg183Trp, p.Lys222Thr, p.Arg285Ter, p.Arg111Ter, p.S184Pfs*46, p.Arg170Cys, p.I160Ffs*25) in *BCKDHB* gene, 1 variant (p.Arg431Ter) in *DBT* gene. In addition, 4 previously unidentified variants (p.Gly281Arg in *BCKDHA* gene, p.Ser171Pro, p.Gly95Ala and p.Lys222Thr in *BCKDHB* gene) were identified. NGS plus Sanger sequencing detection is effective and accurate for gene diagnosis. Computational structural modeling indicated that these novel variations probably affect structural stability and considered as likely pathogenic variants.

## Introduction

Maple syrup urine disease (MSUD, OMIM # 248600) is a hereditary branched-chain amino acid metabolism disorder caused by branched chain α-ketoacid dehydrogenase multi-enzyme complex (BCKDC). Common clinical manifestations of MSUD are feeding difficulties, epilepsy, mental retardation, ketonuria and maple-like body odor. Without timely intervention, the disease progresses rapidly, and the mortality and disability rate are very high. According to the phenotype, MSUD can be divided into 5 types^1^: classic, intermediate, intermittent, thiamine-responsive and dihydrolipoylamide dehydrogenase (E3) deficiency. The classic type is the most common and severe type in the neonatal period, accounting for 75% affected infants. It usually occurs 4–9 days after birth. The liver BCKDC activity in classic type children is often lower than 2% in healthy children, manifested as ketoacidosis, neurologic damage and mental retardation^2^. 20% are intermediate or intermittent type, the intermediate type usually shows a continuously increased concentration of branched-chain amino acids (BCAAs), accompanied by nervous system damage^3^. Intermittent type usually occurs from 5 months to 2 years with mild symptoms^4^, prognosis of the thiamine-responsive type is better than classic type, with BCKDA activity of 2% -40%, and some children can survive for a long time^5^. The activity of dihydrolipoamide acyl dehydrogenase (E3) -deficient BCKDA is 25% lower than that of normal children, and it is characterized by low tension, stunting, and lactic acidosis^6^.

According to the Guidelines for the Diagnosis and Treatment of Maple syrup urine disease (2019 Edition, China), MSUD is diagnosed based on the typical clinical manifestations such as nervous system injury and urine maple sugar odor, elevated plasma level of BCAAs (leucine, isoleucine, and valine) and allo-isoleucine and elevated urine level of branched-chain hydroxyacids and ketoacids(BCKAs). Isoleucine > 5 μmol/L detected by plasma amino acid analyzer can be clinically diagnosed as MSUD. Increased isoleucine and alloisoleucine are the gold standard for diagnosis, and 50% of patients have leucine levels exceeding 1500 μmol/L at the time of diagnosis. Determination of BCKAD complex enzyme activity and variation analysis of four genes (*BCKDHA*, *BCKDHB*, *DBT* and *DLD*) are helpful for definite diagnosis. Tandem mass spectrometry (MSMS) was used to analyze the blood amino acid profile, with leucine, isoleucine and valine levels as the main indicators and leucine/phenylalanine and valine/phenylalanine ratios as the secondary indicators. Those who screened positive were further diagnosed by urine organic acid, plasma amino acid and gene analysis.

MSUD is inherited in autosomal recessive pattern, and it is very rare in most populations, with an incidence of 1:185,000^7^. BCKDC is located in the mitochondrial inner membrane and consists of 4 subunits: Elα, E1β, E2, E3, which are encoded by *BCKDHA*, *BCKDHB*, *DBT* and *DLD* genes, respectively^8^. According to the involved subunit, MSUD is divided into the following types: (1) type Ia (OMIM 608348), caused by biallelic pathogenic variants in *BCKDHA* gene encoding the Elα subunit (2) type I b (OMIM 248611), caused by biallelic pathogenic variants in *BCKDHB* gene encoding the E1β subunit (3) type II (OMlM 248610), caused by biallelic pathogenic variants in *DBT* gene encoding E2 subunit (4) type III (OMIM 238331), caused by biallelic pathogenic variants in *DLD* gene encoding E3 subunit^7^. Another two subtypes Type IV and type V are specific kinase and phosphatase gene mutation types, respectively.

MSUD is a genetically heterogeneous disease, and the traditional sequencing technology is time-consuming and costly. High-throughput sequencing technology based on target gene capture for sequencing of the four genes can simultaneously detect gene mutations in the causative genes, not only providing accurate genetic diagnosis results for patients, but also providing clinicians with the basis for differential diagnosis, drug treatment, subsequent genetic counseling, and prenatal diagnosis. In this study, we applied targeted high-throughput sequencing to sequence the target regions of *BCKDHA*, *BCKDHB*, *DBT* and *DLD* genes in peripheral blood samples of patients or parents in 8 families with MSUD, and Sanger sequencing validation was subsequently performed for confirmation of suspected pathogenic variants.

## Methods

### Subjects

**Figure 1:**
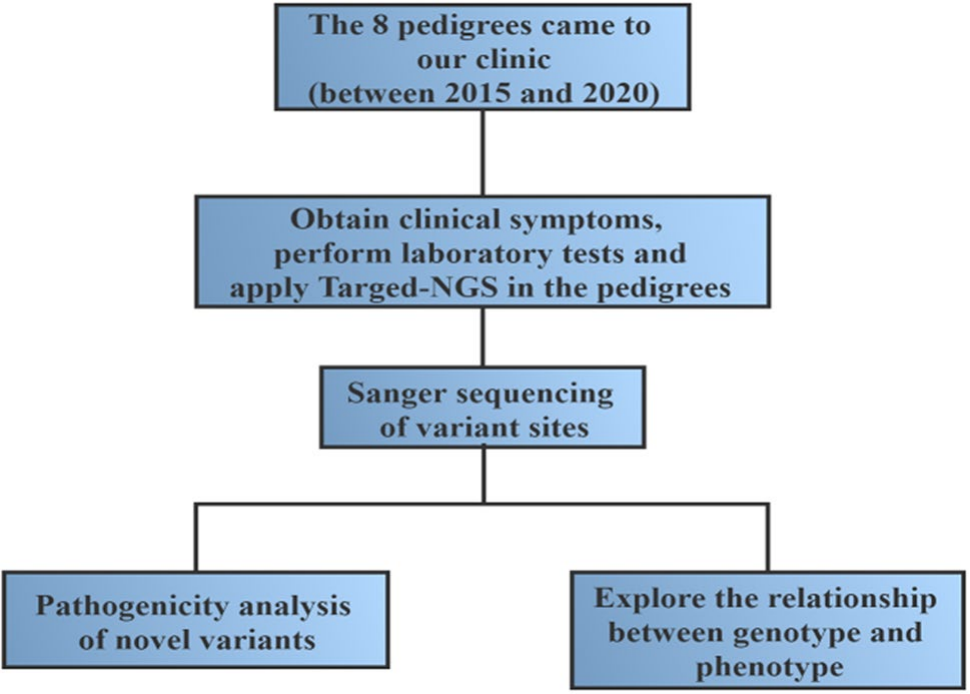
Flowchart of study design of the research.

This is a retrospective study of clinical cases from the First Affiliated Hospital of Zhengzhou University between 2015 and 2020. Eight unrelated families of Chinese Han nationality that had given birth to children affected with MSUD were collected from a single center (Fig. 1). Written informed consent was obtained from the legal guardians. All of the procedures and informed consent were approved by the Medical Ethics Committee of the First Affiliated Hospital of Zhengzhou University (KS-2018-KY-36), and were performed according to the principles of the Declaration of Helsinki.

### Blood amino acid and ester acylcarnitine spectra analysis

Urine and venous blood was collected from the children on an empty stomach for more than 4 h. Urinary organic acid analysis was performed using gas chromatography mass spectrometry (GC–MS) and blood amino acid and ester-acylcarnitine profiling was performed using liquid chromatography-tandem mass spectrometry (LC–MS/MS).

### DNA extraction

Blood samples (2 ml) were collected from each patient and their parents in families 3 and 7 by venipuncture in EDTA tubes. For the remaining six families, parental blood samples were collected. Genomic DNA was extracted from peripheral blood leukocytes using a DNA Blood Mini Kit (Qiagen, Cat.No.51106, Germany) according to the recommended protocol.

### Targeted next-generation sequencing

Targeted genes were chosen according to OMIM database (https://omim.org/) and were designed by the MyGenostics company (Beijing, China). Metabolic disease gene panel was specifically captured and enriched using array-based hybridization chip (NimbleGen, Madison, USA) followed by HiSeq2000 (Illumina, San Diego, USA) sequencing to generate paired read 100 bp according to the manufacturer’s protocol. Then, the final products were amplified by PCR and validated using the Agilent Bioanalyzer. Fastq-format reads were aligned to the human reference genome (GRCh37/hg19, https://genome.ucsc.edu/) using BWA^9^ software (Burrows Wheeler Aligner). Base quality score recalibration together with SNP and short Indel calling was conducted with GATK 3.8^10^. Quality metrics were evaluated the average depth was 80 × per sample, with at least 97% of the target region covered by 10 × reads or more. The VCF files were then annotated using SnpEff^11^. Variants with > 1% frequency in the population variant databases-1000 Genomes Project^12^, Exome Variant Server (EVS, http://evs.gs.washington.edu/EVS/) and Exome Aggregation Consortium (ExAC, http://exac.broadinstitute.org) or > 5% frequency in the local database with 150 exome datasets were filtered, and subsequently intergenic, intronic, and synonymous variants were filtered, except those located at canonical splice sites. By searching the Human Gene Mutation Database (HGMD, http://www.hgmd.org/) to clarify whether the variant is a known pathogenic variant. The nomenclature of new variants was based on the international gene variant nomenclature system (http://www.hgvs.org/mutnomen).

### Validation tests of Sanger sequencing

Gene tool software was used for designing primers for suspected variants. Routine PCR reactions were performed. PCR products were purified and directly sequenced on ABl3130-xl gene sequencing instrument using the ABIBigDye3.1 sequencing kit (Thermo Fisher Scientific, USA), and the sequencing data were compared and analyzed using ABI Sequencing Analysis 5.1.1 software.

### In silico webservers and structure prediction

Multiple sequence alignments were performed using HomoloGene database (http://www.ncbi.nlm.nih.gov/homologene) to verify the degree of conservation. The pathogenicity of the variants was then evaluated using three in silico webservers, PolyPhen2 (http://genetics.bwh.harvard.edu/pph2/), SIFT (http://provean.jcvi.org/index.php) and Mutation Taster (http://www.mutationtaster.org). The American College of Medical Genetics and Genomics (ACMG) guideline was applied to assess novel variants’ pathogenicity. Computational modeling was carried out to observe the effect of new missense variants on protein structure. Three-dimensional structure of the target protein sequence was constructed using PyMOL protein model structure simulation software to determine the effect of amino acid substitution on protein structure.

### Summary of published data

A literature search by using the PubMed and WanFang databases were conducted to identify reported variants in Chinese MSUD patients.The searches were using the Keywords “Maple syrup urine disease (MSUD)” or “*BCKDHA*” or “*BCKDHB*” or “*DBT*” or “*DLD*” and “Chinese”.

## Results

### Characteristics of recruited subjects

Between 2015 and 2020, a total of 8 families were collected in our study. The study design is shown in Fig. 1. Characteristics of these cases are shown in Table 1. Only children in family 3 and 7 accepted timely diagnosis and treatment after neonatal screening. All children in the 8 families were screened by tandem mass spectrometry and received positive screening results. As is shown in Table 1, the remaining 6 children developed the disease from 3 days to 4 months, and died at 16 days, 2 months, 20 days, 1 month, 10 days and 1 month, respectively.

**Table 1:** Clinical and laboratory features of the 8 patients with MUSD.

Patient ID	Sex	Age of onset	Age of diagnosis	Clicinal subtype	Clicinal symptoms	Blood tandem mass spectrometry	GC–MS organic acids	Cranial CT/MRI	Follow up outcome
P1	F	7d	10d	Classic	Vomiting Poor response Coma	Leu/Ile-1904 Val-354 Leu/Phe-34.2	MSUD pattern	NA	Died at 16d
P2	F	1 m	1 m	Intermediate	Convulsion Feed difficulties Hypermyotonia Maple syrup odor	Leu/Ile-935 Val-295 Leu/Phe-19.4	MSUD pattern	Extensive Low-density changes in the brain	Died at 2 m
P3	M	4 m	4 m	Intermediate	Mental retardation Motor development delay	Leu/Ile-1122.88 Val-325.05 Leu/Phe-29.92	MSUD pattern	Bilateral Frontotemporal parietal white mater density symmetry decreased	Mental retardation
P4	F	10d	12d	Intermediate	Vomiting Coma Feed difficulties	Leu/Ile-679 Val-420 Leu/Phe-12.6	NA	NA	Died at 20d
P5	M	4d	10d	Classic	Poor response Feed difficulties Seizure	Leu/Ile-3089 Val-578 Leu/Phe-38.2	MSUD pattern	Extensive hypodensity changes in bilateral ventricles	Died at 1 m
P6	M	3d	6d	Classic	Poor response Convulsion Feed difficulties Hypermyotonia Maple syrup odor	Leu/Ile-3209 Val-521 Leu/Phe-39.1	NA	NA	Died at 10d
P7	F	17d	25d	Intermediate	Poor response Convulsion Feed difficulties Hypermyotonia	Leu/Ile-1754 Val-302.92 Leu/Phe-22	MSUD pattern	Poor myelination in white matter, diffuse long T1 and long T2 Edema signal changes in subcortical white matter and frontotemporal white matter	Mental retardation
P8	M	5d	10d	Classic	Poor response Convulsion Feed difficulties	Leu/Ile-2748 Val-520 Leu/Phe-29.5	MSUD pattern	NA	Died at 1 m

*F* Female, *M* Male, *d* day, *m* month, *Leu* Leucine, *Ile* Ileucine, *Val* Valine, *Phe* Phenylalanine, *TMS* acylcarnitine analysis using tandem mass spectrometry, *GC–MS* urinary arganic acid analysis Gas Chromatography mass spectrometry, *NA* not available.

MSUD pattern: increased levels of ketoacids: 2-hydroxy isovaleric acid, 2-keto isovaleric acid, 2-keto-3-methylpentanoic acid and 2-keto-isohexanoic acid.

Normal ranges on TMS: Leu/Ile < 350 μmol/L, Val < 250 μmol/L, Leu/Phe < 8.

### Molecular analysis in *BCKDHA*, *BCKDHB* and *DBT* genes

NGS was performed in children of families 3 and 7 and couples of the other 6 MSUD families to detect the sequence variation in each exon of the 4 causative genes (*BCKDHA*, *BCDKHB*, *DBT*, *DLD*) associated with MSUD. After alignment with the hg19 sequence, the variants were filtered by excluding the SNPs (normal frequency > 0.05) reported in the dbSNP137 database, Hapmap database and 1000 Genome database. Suspected variants in the causative genes associated with the 8 families were shown in Table 2.

**Table 2:** Detection and analysis of gene variants in 8 MSUD families.

Patient ID	Gene	Proband genotype		Prediction software analysis				Pathogenicity assessment based on ACMG guideline
		Genotype 1/paternal genotype	Genotype 2/maternal genotype	SIFT	Polyphen-2	MutationTaster	Conservation	
P1	*BCKDHA*	c.841G>C(p.Gly281Arg)^a^	c.841G>C(p.Gly281Arg)^a^	Damaging	Probably damaging	Disease causing	Conserved	PM3 + PP3 + PP4
P2	*BCKDHA*	c.647C>T(p.Ala216Val)	c.647C>T(p.Ala216Val)					
P3	*BCKDHB*	c.508C>T(p.Arg170Cys)	c.511T>C(p.Ser171Pro)^a^	Damaging	Probably damaging	Disease causing	Conserved	PM3 + PP3 + PP4
P4	*BCKDHB*	c.547C>T(p.Arg183Trp)	c.665A>C(p.Lys222Thr)^a^	Damaging	Probably damaging	Disease causing	Conserved	PM3 + PM5 + PP3 + PP4
P5	*BCKDHB*	c.284G>C(p.Gly95Ala)^a^	c.853C>T(p.Arg285Ter)	Damaging	Probably damaging	Disease causing	Conserved	PM3 + PP3 + PP4
P6	*BCKDHB*	c.331C>T(p.Arg111Ter)	c.550delT(p.S184Pfs*46)					
P7	*BCKDHB*	c.523T>C(p.Phe175Leu)	c.478-552del(p.I160Ffs*25)					
P8	*DBT*	c.1291C>T(p.Arg431Ter)	c.1291C>T(p.Arg431Ter)					

^a^Novel variation.

### Sanger sequencing results

**Figure 2:**
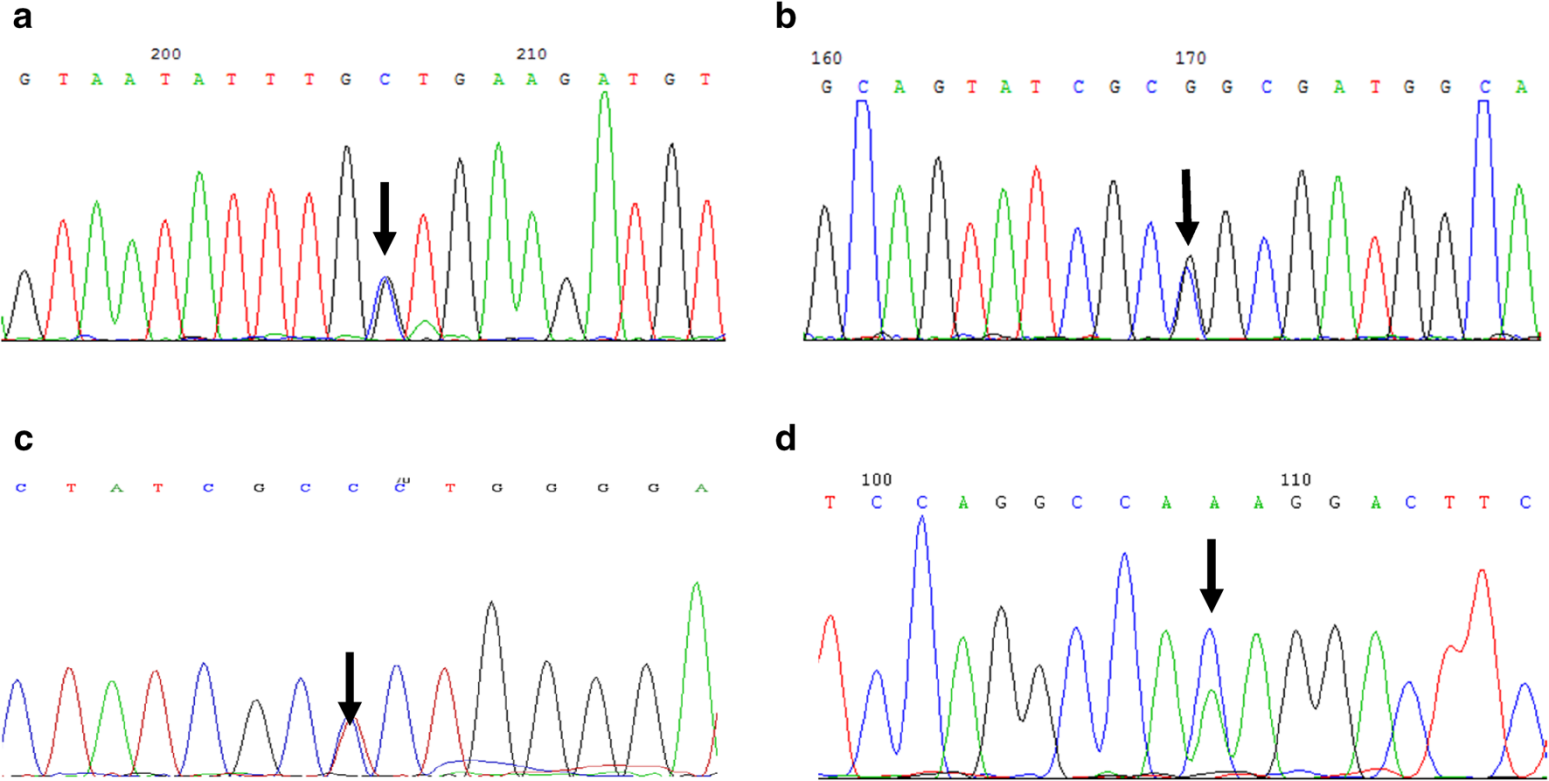
Gene sequences of four novel variants in *BCKDHA* and *BCKDHB* genes in eight pedigrees: c.841G>C(p.Gly281Arg) in *BCKDHA* gene (**a**), c.284G>C(p.Gly95Ala) in *BCKDHB* gene (**b**), c.511T>C(p.Ser171Pro) in *BCKDHB* gene (**c**), c.665A>C(p.Lys222Thr) in *BCKDHB* gene (**d**).

The suspected variants found by NGS were confirmed by Sanger sequencing. The patient in family 3 carried *BCKDHB* gene c.511T>C(p.Ser171Pro) and c. c.508C>T(p.Arg170Cys) compound heterozygous variants, and the child in pedigree 7 carried c.523T>C(p.Phe175Leu) and c.478-552del(p.I160Ffs*25) compound heterozygous variants. Their parents were heterozygous carriers of the respective variant. Heterozygous variants in the same causative gene of MSUD were detected in both couples in the remaining six families. Gene sequences of four novel variant in *BCKDHA* and *BCKDHB* genes were shown in Fig. 2.

### In silico prediction of novel gene variant sequences

Mutation Taster and PolyPhen-2 analysis showed that four novel missense variants: p.Gly95Ala, p.Gly281Arg, p.Lys222Thr and p.Ser171Pro were highly likely to be pathogenic/deleterious variants. We use The American College of Medical Genetics and Genomics (ACMG)^13^ guideline to assess these novel variants’ pathogenicity in Table 2.

### Three-dimensional structure of proteins

**Figure 3:**
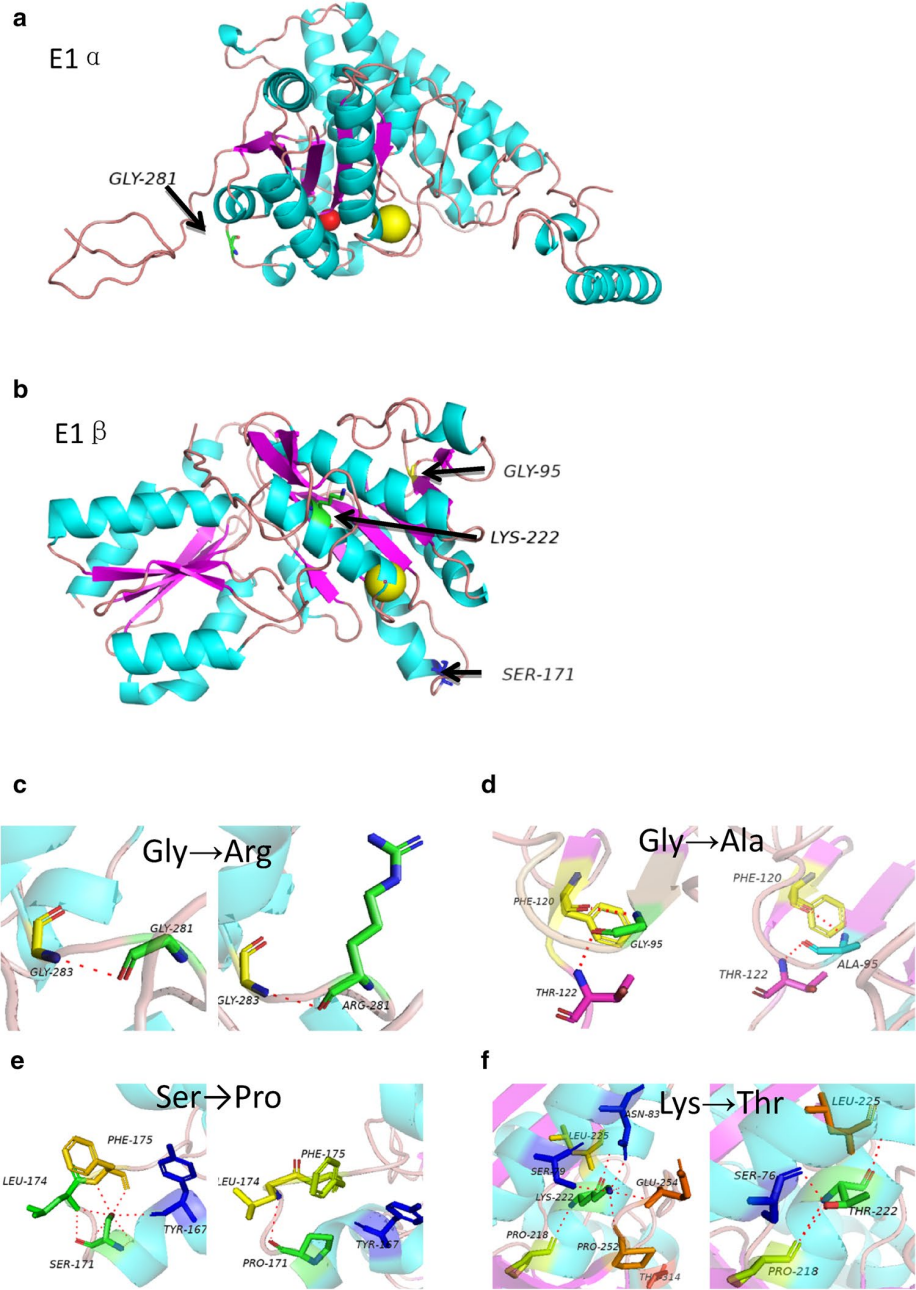
(**a**) Predicted 3D protein structure of E1α compotent complex and the site of the Glycine (Gly281) colored in green. (**b**) Predicted 3D protein structure of E1β compotent complexand the site of three Residues. The Glycine (Gly95), Lysine (Lys222), Serine (Ser171) are colored yellow, green and blue respectively. Predicted protein structures of four novel variants in *BCKDHA* and *BCKDHB* genes: p.Gly281Arg (**c**) in *BCKDHA* gene, p.Gly95Ala (**d**), p.Ser171Pro (**e**) and p.Lys222Thr (**f**) in *BCKDHB* gene.

The predicted three-dimensional structures of 4 novel variants in *BCKDHA* and *BCKDHB* genes were shown in Fig. 3. In *BCKDHA* gene, Glycine 281 is located in the random coil structure of protein secondary structure. Glycine lacks side chain (only one H-bond). After variation to arginine, arginine is a basic amino acid with ions, affecting the stability of E1α tertiary structure, thus affecting protein function.

In *BCKDHB* gene, As is shown in Fig. 3d, Gly95 is located in the β-turn region, and due to the lack of side chains (only one H atom) by Glycine, there is no steric hindrance, allowing a U-shaped turn of the peptide chain by 180° in the β-turn. Variation of Glycine to Alanine, which forms some steric hindrance, is likely to change the turning of the β-turn, causing change in local steric conformation.

In Fig. 3e, Serine at position 171 is located in the α-helix of protein secondary structure, forming hydrogen bonds with Leucine at position 174, Phenylalanine at position 175 and Tyrosine at position 167. After variation to Proline, the hydrogen bonds with Leucine at position 167 and Phenylalanine at position 175 disappear, affecting the stability of protein secondary structure. Therefore, it is speculated that p.Ser171Pro variant has a greater impact on protein function.

In Fig. 3f, Amino acid 222 is located in the α-helix of the secondary structure of the protein, amino acid 222 forms hydrogen bonds with amino acids 79, 83, 218, 225, 252, and 254. After variation to Threonine, it reforms hydrogen bonds with amino acids 76, 218, and 225. The secondary structure of the protein is changed, which disrupts the stability of the protein and may affect the cleavage and activation function of the protein.

### Published data analysis

Collectively, 15 studies were included for analysis of genotype and phenotype (Supplementary Table S1). In the combined data with ours, there were 61 cases in reported Chinese patients, of which 17 (27.9%) cases had *BCKDHA* gene variations, 35 (57.4%) cases had *BCKDHB* gene variations, 9 (14.7%) cases carried *DBT* gene variations. And no patients carried *DLD* gene variations. According to the clinical manifestations and laboratory tests, Forty-five (73.4%) were diagnosed as classic type (13/*BCKDHA*, 25/*BCKDHB*, 7/*DBT*), 1 the intermittent type (1/*BCKDHA*), 9 the intermediate type (2/*BCKDHA*, 7/*BCKDHB*), 4 the thiamine-responsive type (1/*BCKDHA*, 1/*BCKDHB*, 2/*DBT*). We counted variants with frequency number > 2 in Chinese patients (Table 3).

**Table 3:** Variants with frequency number > 2 in reported Chinese patients.

Gene	Nucleotide change	Amino acid change	Number	%
*BCKDHB*	c.331C>T	p.Arg111Ter	7	9
*BCKDHB*	c.853C>T	p.Arg285Ter	6	7
*BCKDHB*	c.508C>T	p.Arg170Cys	3	4
*BCKDHB*	c.659delA	p.Asp229fs	3	4
*DBT*	c.75-76delAT	p.Cys26Trpfs*2	3	4
*DBT*	c.1291C>T	p.Arg431Ter	3	4

## Discussion

Maple syrup urine diabetes is a branched-chain amino acid metabolism disease caused by mitochondrial branched-chain α-keto acid dehydrogenase (BCKDC) deficiency. Scaini et al.^14^ suggested that cognitive impairment after accumulation of branched-chain amino acids is mainly due to oxidative damage to the brain. The clinical manifestations of MSUD are lack of specificity with rapid onset. The detection of amino acid levels and the ratio between related amino acids in hemofilter paper by tandem mass spectrometry^15^ allow for early screening of MSUD and provide an important basis for further diagnosis and treatment. In this study, the results of blood tandem mass spectrometry in all families showed that both leucine and valine were significantly higher, accompanied by amino acid ratio changes, consistent with MSUD biochemical findings.

In our study, a total of 13 variants (15.4% located in the *BCKDHA* gene, 76.9% in the *BCKDHB* gene, and 7.7% in the *DBT* gene, no variants in *DLD* gene) were identified in 16 alleles in 8 families. In the systematic literature review of MSUD reported in Chinese population, 81 mutations have been detected in 61 patients in China, including the 8 patients in our study. There are 26 (32.1%) gene variants located in the *BCKDHA* gene, 45 (55.6%) gene variants in the *BCKDHB* gene, 10 (12.3%) gene variants in the *DBT* gene, no variants in the *DLD* gene. The *BCKDHB* gene may be a major variant type of MSUD in the Chinese population. Gene variations of MSUD patients are mainly concentrated in the *BCKDHB* gene, followed by *BCKDHA* and *DBT* genes^16^. Current study suggested that *DLD* gene variants account for 13%^17^. While our data are inconsistent with this, *DLD* gene variants may be very rare in Chinese population. MSUD gene has high allelic heterogeneity, with the exception of gene mutation hotspots found in minority of ethnic groups, such as the most common mutation in the Mennonite community being the *BCKDHA* gene c.1312T>A (p.Tyr393Asn)^18^, Portuguese gypsy mutation hotspot c.117delC^19^. The *BCKDHB* gene c.538G>C was a common mutation found in Ashkenazi Jews^20^, and exon 5 of the *BCKDHB* gene may be a region of genetic variation and a hotspot region^21,22^. Hotspot mutations are not found in the remaining population^23–25^. There were no significant hotspot mutations have been identified in the Chinese population^26–32^. Variants c.331C>T and c.853C>T in *BCKDHB* gene may be relatively common in Chinese patients.

Four variants were novel variants, one located in *BCKDHA* gene (p. Gly281Arg) and three located in *BCKDHB *gene (p. Gly95Ala, p.Lys222Thr, p.Ser171Pro) illustrates that the disease has high allelic heterogeneity. Protein structure prediction was carried out for BCKDHA, BCKDHB in order to analyze the variants in a visual way. In BCKDHA, variation p. Gly281Arg is present in coil and minimal change has been discovered in the protein structure. In BCKDHB, p. Gly95Ala is present in the β-turn region, which may change the turning of the β-turn and causing change in local steric conformation. Two variants (p.Lys222Thr and p.Ser171Pro) are present in the α-helix of the secondary structure of the protein and thus have great impact on the protein function. According to the American College of Medical Genetics and Genomics (ACMG) standard and guidelines, the three variants (p.Gly281Arg of *BCKDHA*, p.Gly95Ala and p.Ser171Pro of *BCKDHB*) are variants of uncertain significance (PM3 + PP3 + PP4), and p.Lys222Thr of *BCKDHB* is likely pathogenic (PM3 + PM5 + PP3 + PP4). All the novel variants are predicted to be disease causing by prediction software (PolyPhen2, SIFT and Mutation Taster). In summary, these four novel variant may be causative variants.

The relationship between MSUD genotype and phenotype has not yet been established. The incidence of the disease is low, and fewer cases are included in each study, making it difficult to obtain an exact genotype–phenotype relationship. Current studies suggest that patients with *BCKDHA* and *BCKDHB* gene variants mostly present with classical type, BCKDH activity is less than 2%. *DBT* gene variants accounts for about 24%, and most of them are thiamine responsive type^6^. The clinical manifestations of patients are relatively mild, including developmental retardation and hypotonia. Patients with nonsense variations presented the severe classic phenotype. Variations in p.Arg111Ter and p.Arg285Ter in *BCKDHB* gene generate premature termination codons and the encoded protein has serious effects on the activity of the complex^33^. Our cases in family 5 and 6 carry the nonsense variants p.Arg111Ter and p.Arg285Ter, respectively, and they have classic phenotype. However, the same type of genetic variation also leads to different clinical phenotypes. For example, In *BCKDHA* gene, p.Glu327Lys has been reported to be associated with intermediate phenotype^8^ while the same variation results in classic phenotype in patients in another study^22^. In our study, patient 4 carries the missense variation p.Arg183Trp in the *BCKDHB* gene and shows intermediate phenotype, while the patient who had the same variation showed classic phenotype in a previous report^34^. Therefore, we could not establish any genotype–phenotype correlation in our patients with MSUD. Half of our cases are classical phenotypes and half are intermediate phenotypes. Majority of patients with intermediate phenotype had variants in the *BCKDHB* gene. All the three genes are implicated in classic phenotype. Most of Chinese patients carried *DBT* gene variations were diagnosed as classic type, while in Norway^35^, patients had *DBT* gene variants were intermittent phenotype. All patients with classic phenotype have worst clinical outcome.

MSUD is a fatal and disabling inherited metabolic disease which is difficult to treat, and has a poor prognosis. Untreated classical children mostly die shortly after birth. The principle of treatment is to remove the inducement, reduce the toxic effect of blood leucine, correct acute metabolic disorders, maintain plasma branched-chain amino acids in the ideal range, and ensure good nutrition and growth and development^36^. MSUD treatment mainly includes acute phase management, dietary management and vitamin B1 treatment. In recent years, liver transplantation for MSUD has been reported^37,38^. However, shortage of liver sources, high cost, and the need to take immunosuppressive agents for a long time after surgery are disadvantages of this treatment. Currently, the best preventive strategy for the disease is to avoid the birth of affected children through prenatal diagnosis. When MUSD is clinically suspected, capture-based high throughput sequencing followed by Sanger sequencing confirmation allows for accurate detection of gene mutations in the causative genes in an effective manner.

In conclusion, we present the clinical characteristics and 16 variants in 8 patients with MSUD and explore the genotype–phenotype relationship. We identified four pathogenic variants in the *BCKDHA* and *BCKDHB* gene by applying high throughput sequencing technology based on target gene capture for sequencing, which have not been previously reported in the Chinese population. This article will contribute to a better understanding of the MSUD variation spectrum identified so far. NGS combined with Sanger sequencing can detect gene variants in the causative genes in an effective way, providing clinicians with the basis for differential diagnosis, drug treatment, subsequent genetic counseling and prenatal diagnosis.

## Supplementary Information

Supplementary Information.
