# Author Correction: Genetic analysis by targeted next-generation sequencing and novel variation identification of maple syrup urine disease in Chinese Han population

**DOI:** 10.1038/s41598-021-00718-4

**Published:** 2021-10-20

**Authors:** Xiaohua Fang, Xiaofan Zhu, Yin Feng, Ying Bai, Xuechao Zhao, Ning Liu, Xiangdong Kong

**Affiliations:** Obstetrics and Gynecology Department, Genetics and Prenatal Diagnosis Center, The First Affiliated Hospital of Zhengzhou University, Zhengzhou, 450052 People’s Republic of China

Correction to: *Scientific Reports* 10.1038/s41598-021-98357-2, published online 23 September 2021

The original version of this Article contained an error in the order of the author names, which was incorrectly given as Xiaohua Fang, Xiaofan Zhu, Yin Feng, Ying Bai, Xuechao Zhao, Xiangdong Kong & Ning Liu.

The original Article has been corrected.

