# Supplementary material for: Genetic analysis by targeted next-generation sequencing and novel variation identification of maple syrup urine disease in Chinese Han population
Source: Sci Rep. 2021 Sep 23;11:18939. doi: 10.1038/s41598-021-98357-2 (PMC8460745; doi:10.1038/s41598-021-98357-2)
Supplement: Supplementary file 1 — Supplementary Information. [file 41598_2021_98357_MOESM1_ESM.pdf]

Title：Genetic analysis by targeted next-generation sequencing and novel variation identification of maple syrup urine disease in Chinese Han population

Author：Corresponding author:

Ning Liu E-mail: liuning903@163.com

The First Affiliated Hospital of Zhengzhou University CN

Xiaohua Fang E-mail: 593744717@qq.com

The First Affiliated Hospital of Zhengzhou University CN

Xiaofan Zhu E-mail: 13838046749@126.com

The First Affiliated Hospital of Zhengzhou University CN

Yin Feng E-mail: fengyin316@163.com

The First Affiliated Hospital of Zhengzhou University CN

Ying Bai E-mail: baiying198611@126.com

The First Affiliated Hospital of Zhengzhou University CN

Xuechao Zhao E-mail: zxc832186@163.com

The First Affiliated Hospital of Zhengzhou University CN

Xiangdong Kong E-mail: kongxd@263.net

The First Affiliated Hospital of Zhengzhou University CN

Supplementary Table 1 Description of reported gene variants in Chinese patients

Patient	Gene	Genotype 1	Genotype 2	Clicinal subtype	Reference
1	*BCKDHA*	c.308T>C(p.Leu103Pro)	c.794G>C(p.Arg265Pro)	classic	1
2	*BCKDHA*	c.863G>A(p.Gly288Asp)	Exon6-8del	classic	2
3	*BCKDHA*	c.349C>T(p.Arg117Cys)	c.562G>T(p.Gly188Trp)	classic	3
4	*BCKDHA*	c.859C>G(p.Arg287Gly)	c.1270dupC(p.Gln424Profs*22)	classic	3
5	*BCKDHA*	c.117dupC(p.Arg40Glnfs*11)	c.565C>T(p.Arg189Cys)	intermediate	3
6	*BCKDHA*	c.55C>T(p.Gln19Ter)	c.452C>T(p.Thr151Met)	intermittent	3
7	*BCKDHA*	c.659C>T(p.Ala220Val)	c.808G>A(p.Ala270Thr)	Thiamine-responsive	3
8	*BCKDHA*	c.740A>G(p.His247Arg)	IVS6+1delG	classic	4
9	*BCKDHA*	c.659C>T(p.Ala220Val)	/	classic	4
10	*BCKDHA*	c.178G>T(p.Glu60Ter)	c.1214_1219dupCCAACC(p.Pro405_Asn406dup)	classic	4
11	*BCKDHA*	c.178G>T(p.Glu60Ter)	c.491T>C(p.Leu164Pro)	classic	4
12	*BCKDHA*	c.539G>A(p.Gly180Asp)	c.794G>A(p.Arg265Gln)	classic	5
13	*BCKDHA*	c.841G>C(p.Gly281Arg)#	c.841G>C(p.Gly281Arg)#	classic	This study
14	*BCKDHA*	c.647C>T(p.Ala216Val)	c.647C>T(p.Ala216Val)	intermediate	This study
15	*BCKDHA*	c.868G>A(p.Gly290Arg)	/	classic	6
16	*BCKDHA*	c.308T>C(p.Leu103Pro)	c.562G>T(p.Gly188Trp)	classic	6
17	*BCKDHA*	c.1279C>G(p.Leu427Val)	c.1280-1291del12(p.Leu427LeufsTer20)	classic	6
18	*BCKDHB*	c.304G>A(p.Gly102Arg)	c.331C>T(p.Arg111Ter)	classic	7
19	*BCKDHB*	c.90_91insCTGGCGCGGGG (p.Phe35TrpfsTer41)	c.80_90del(p.Ala32PhefsTer48)	classic	8
20	*BCKDHB*	c.580C>T(p.Leu194Phe)	c.597T>C(p.Ser199Arg)	classic	9
21	*BCKDHB*	c.509G>A(p.Arg170His)	c.1037A>G(p.Gln346Arg)	classic	10
22	*BCKDHB*	c.508C>T(p.Arg170Cys)	c.659delA(p.Asp229fs)	classic	2
23	*BCKDHB*	c.523T>C(p.Phe175Leu)	Exon1-7del	intermediate	2
24	*BCKDHB*	c.659delA(p.Asp229fs)	c.659delA(p.Asp229fs)	classic	2
25	*BCKDHB*	c.93-103dup11(p.Phe35fs)	c.550delT(p.Ser184fs)	classic	2
26	*BCKDHB*	c.275-2A>G	c.275-2A>G	classic	3
27	*BCKDHB*	c.853C>T(p.Arg285Ter)	c.853C>T(p.Arg285Ter)	classic	3
28	*BCKDHB*	c.331C>T(p.Arg111Ter)	c.331C>T(p.Arg111Ter)	classic	3
29	*BCKDHB*	c.391G>A(p.Gly131Arg)	c.1006G>A(p.Gly336Ser)	classic	3
30	*BCKDHB*	IVS4-2A>C	IVS4-2A>C	classic	4
31	*BCKDHB*	c.767A>G(p.Tyr256Cys)	c.768C>G(p.Tyr256Ter)	intermediate	4
32	*BCKDHB*	c.482T>G(p.Val161Gly)	c.508C>T(p.Arg170Cys)	unknown	4
33	*BCKDHB*	c.506A>G(p.Tyr169Cys)	c.93_103del11(p.Ala32PhefsTer48)	classic	5
34	*BCKDHB*	c.991G>A(p.Ala331Thr)	c.1006G>A(p.Gly336Ser)	intermediate	5
35	*BCKDHB*	c.331C>T(p.Arg111Ter)	c.715_716GA>TT(p.Glu239Leu)	classic	6
36	*BCKDHB*	c.853C>T(p.Arg285Ter)	c.853C>T(p.Arg285Ter)	classic	6
37	*BCKDHB*	c.593A>T(p.Gln198Leu)	c.1046G>A(p.Cys349Tyr)	intermediate	6
38	*BCKDHB*	c.230T>C(p.Val77Ala)	c.230T>C(p.Val77Ala)	Thiamine-responsive	6
39	*BCKDHB*	c.853C>T(p.Arg285Ter)	c.951+1G>T	classic	6
40	*BCKDHB*	c.383G>A(p.Gly128Glu)	c.1028delC(p.Ser343LeufsTer9)	classic	6
41	*BCKDHB*	c.331C>T(p.Arg111Ter)	c.331C>T(p.Arg111Ter)	classic	6

42	*BCKDHB*	c.410C>T(p.Ala137Val)	/	classic	[6]
43	*BCKDHB*	c.508C>T(p.Arg170Cys)	c.511T>C(p.Ser171Pro)#	intermediate	This study
44	*BCKDHB*	c.547C>T(p.Arg183Trp)	c.665A>C(p.Lys222Thr)#	intermediate	This study
45	*BCKDHB*	c.284G>C(p.Gly95Ala)#	c.853C>T(p.Arg285Ter)	classic	This study
46	*BCKDHB*	c.331C>T(p.Arg111Ter)	c.550delT(p.S184Pfs*46)	classic	This study
47	*BCKDHB*	c.523T>C(p.Phe175Leu)	c.478-552del(p.I160Ffs*25)	intermediate	This study
48	*BCKDHB*	c.254T>C(p.Leu85Ser)	c.254T>C(p.Leu85Ser)	classic	[11]
49	*BCKDHB*	c.517G>A(p.Asp173Tyr)	c.517G>A(p.Asp173Tyr)	classic	[12]
50	*BCKDHB*	c.503G>A(p.Arg168His)	c.503G>A(p.Arg168His)	classic	[12]
51	*BCKDHB*	c.391G>A(p.Gly131Arg)	Chr6:g.80811266-81194921del383556	classic	[13]
52	*BCKDHB*	c.1076G>A(p.Arg359Lys)	c.705delT(p.Cys235Ter)	unknown	[13]
53	*DBT*	c.75-76delAT(p.Cys26Trpfs*2)	c.75-76delAT(p.Cys26Trpfs*2)	classic	[3]
54	*DBT*	c.75-76delAT(p.Cys26Trpfs*2)	c.1291C>T(p.Arg431Ter)	classic	[3]
55	*DBT*	c.1A>G(p.Met1Val)	c.1A>G(p.Met1Val)	classic	[4]
56	*DBT*	c.206T>G(p.Leu69Arg)	c.871C>T(p.Arg291Ter)	classic	[5]
57	*DBT*	c.51+5G>C	c.768delA(p.Ile256IlefsTer15)	Thiamine-responsive	[6]
58	*DBT*	c.372_377del6(p.Asn124Asnfs)	c.372_377del6(p.Asn124Asnfs)	classic	[6]
59	*DBT*	c.713delC(p.Pro238LeufsTer3)	c.713delC(p.Pro238LeufsTer3)	classic	[6]
60	*DBT*	c.1132C>T(p.378X)	c.1132C>T(p.378X)	Thiamine-responsive	[15]
61	*DBT*	c.1291C>T(p.Arg431Ter)	c.1291C>T(p.Arg431Ter)	classic	This study

References:

1. Wang, J. et al. Identification of Two Novel BCKDHA Mutations in a Chinese Patient with Maple Syrup Urine Disease. *Journal of Pediatric Endocrinology and Metabolism*. **24**, (2011).
2. Li, X. et al. Clinical Characteristics and Mutation Analysis of Five Chinese Patients with Maple Syrup Urine Disease. *Metab. Brain Dis.* **33**, 741-751 (2018).
3. Sun, W. et al. Identification of Eight Novel Mutations in 11 Chinese Patients with Maple Syrup Urine Disease. *World J. Pediatr.* **16**, 401-410 (2020).
4. Li, X. et al. Eleven Novel Mutations of the BCKDHA, BCKDHB and DBT Genes Associated with Maple Syrup Urine Disease in the Chinese Population: Report On Eight Cases. *Eur. J. Med. Genet.* **58**, 617-623 (2015).
5. Yang, C., Linpeng, S., Cao, Y. & Wu, L. Identification of Six Novel Mutations in Five Infants with Suspected Maple Syrup Urine Disease Based On Blood and Urine Metabolism Screening. *Gene*. **710**, 9-16 (2019).
6. Yang, N. et al. Analysis of Gene Mutations in Chinese Patients with Maple Syrup Urine Disease. *Mol. Genet. Metab.* **106**, 412-418 (2012).
7. Li, T. et al.Maple syrup urine disease and gene mutations in twin neonates. *Chin J Contemp Pediatr.***18**, 1242-1246 (2016).
8. Wang, J. et al. Clinical characteristics of maple syrup urine disease and a case of medical nutrition therapy. *Chinese Journal of clinical Nutrition*.**28**, 44-50 (2020).
9. Shen, Y., Gong, X., Yan, J.,Qin L. & Qiu, G. Maple syrup urine disease caused by two novel BCKDHB gene mutations in a Chinese neonate.*Chin J Pediatr.* **53**, 66-70 (2015).
10. Wang, Y., Qi, M., Li, T. & Zhao, Y. Two Novel Mutations in the BCKDHB Gene (R170H, Q346R) Cause the Classic Form of Maple Syrup Urine Disease (MSUD). *Gene*. **498**, 112-115 (2012).

11. Feng, Y., Mai, J., Yang, Z. & Chen S.Homozygous Mutation of BCKDHB Gene in Maple Syrup Urine Disease of Newborns:One Case Report and Literature Review. *Chinese General Practice.* **20**, 2159-2162 (2017).

12. Su, L. et al. Two Homozygous Mutations in the Exon 5 of BCKDHB Gene that May Cause the Classic Form of Maple Syrup Urine Disease. *Metab. Brain Dis.* **32**, 765-772 (2017).

13. Liu, G. et al. A Novel Whole Gene Deletion of BCKDHB by Alu-Mediated Non-Allelic Recombination in a Chinese Patient with Maple Syrup Urine Disease. *Frontiers in Genetics*. **9**, (2018).

14. Liu, Y. et al. Paroxysmal Spasticity of Lower Extremities as the Initial Symptom in Two Siblings with Maple Syrup Urine Disease. *Mol. Med. Rep.* **19**, 4872-4880 (2019).

15. Feng, W., Jia, J., Guan, H. & Tian, Q. Case Report: Maple Syrup Urine Disease with a Novel DBT Gene Mutation. *BMC Pediatr.* **19**, (2019).
